# A comparison of serum and plasma levels of vascular endothelial growth factor during the menstrual cycle in healthy female volunteers

**DOI:** 10.1038/sj.bjc.6600322

**Published:** 2002-06-05

**Authors:** C McIlhenny, W D George, J C Doughty

**Affiliations:** University Department of Surgery, Western Infirmary, Glasgow G11 6NT, UK

**Keywords:** VEGF, breast cancer, timing of surgery

## Abstract

Angiogenesis is the formation of new blood vessels from the existing vasculature, and is essential for the growth and metastasis of most solid tumours. One of the most important growth factors involved in the angiogenesis process is vascular endothelial growth factor. Vascular endothelial growth factor expression has been shown to be regulated by female hormones in breast cancer cell lines, and two previous authors have reported on cyclical variations in serum vascular endothelial growth factor concentrations with conflicting results. No work has been performed on variations in plasma levels of vascular endothelial growth factor during the menstrual cycle. We therefore conducted the first prospective trial to compare serum and plasma levels of vascular endothelial growth factor in healthy pre-menopausal volunteers. Twenty healthy pre-menopausal women were recruited and had blood samples taken over one menstrual cycle with an average of eight samples taken per patient. Plasma and serum samples were then analysed for sex hormones and vascular endothelial growth factor 165. Serum vascular endothelial growth factor levels were found to be significantly higher than plasma vascular endothelial growth factor levels (*P*<0.005). We found no significant difference between serum and plasma vascular endothelial growth factor in the luteal and follicular phases of the cycle. The majority of the measurements for plasma levels of vascular endothelial growth factor at all phases of the cycle were under the limit of detection of the vascular endothelial growth factor ELISA kit. We found no significant correlation between plasma or serum levels of vascular endothelial growth factor and either FSH, LH, Oestradiol or Progesterone levels. This study has demonstrated no difference in serum concentrations of vascular endothelial growth factor during the different phases of the menstrual cycle in a group of healthy volunteers. We also demonstrated no obvious difference in plasma concentrations of vascular endothelial growth factor between the phases of the cycle, but most of the measurements were below the level of accuracy reported by the ELISA kit manufacturer. With the sensitivity of this ELISA test, therefore, we must still regard the question of whether there is a variation in plasma concentrations of vascular endothelial growth factor throughout the menstrual cycle as unanswered.

*British Journal of Cancer* (2002) **86**, 1786–1789. doi:10.1038/sj.bjc.6600322 www.bjcancer.com

© 2002 Cancer Research UK

## 

Angiogenesis is the formation of new blood vessels from the existing vasculature, and is essential for the growth and metastasis of many solid tumours (Folkman, 1990), including breast cancer (Weidner, 1991). The degree of angiogenesis of a tumour is directly related to the aggressiveness of the tumour, and serves as an independent prognostic factor in breast cancer (Bosari, 1992). One of the most important growth factors involved in the angiogenesis process is vascular endothelial growth factor (VEGF). VEGF induces endothelial cell proliferation and it plays a vital role in regulating both physiologic and pathological angiogenesis. VEGF is a heparin binding glycoprotein that is secreted as a homodimer of 45 kDa. Most types of cells, but usually not endothelial cells themselves, secrete VEGF. VEGF can also stimulate cell migration and inhibit apoptosis. Expression of VEGF in breast tumours correlates directly with the degree of angiogenesis of the tumour, and is an independent indicator of nodal metastases and disease free survival (Hitoshi, 1996; Obermair, 1997).

In addition to being upregulated within tumours, various growth factors and angiogenic cytokines, including VEGF, have been demonstrated in the sera and other body fluids of patients with a wide variety of cancers (Yamamoto, 1996). In breast cancer serum levels of VEGF have been shown to rise with increasing stage of disease, and in colorectal cancer serum VEGF levels have been used to predict oncological clearance (Kumar *et al*, 1998).

Vascular endothelial growth factor expression has been shown to be regulated by female hormones in breast cancer cell lines (Hyder *et al*, 1998; Nakamura *et al*, 1996). It has been hypothesised that circulating levels of VEGF may alter with the menstrual cycle and be responsible for the reported increase in recurrence rate in patients operated on during the follicular phase of the cycle. Two recent studies have reported on this hypothesis with conflicting results. Heer *et al* (1998) demonstrated a higher serum level of VEGF during the follicular phase of the cycle in 14 healthy pre-menopausal volunteers. Chung *et al* (1998) however, demonstrated no significant differences in serum VEGF during the two phases of the cycle again in 14 healthy pre-menopausal volunteers.

Recent work has thrown doubt on the validity of measuring serum levels of VEGF. Since these studies have been carried out, it has become apparent that many haematological cells are capable of releasing VEGF, especially platelets (Banks, 1998). The extent to which these cells normally contribute to the circulating VEGF pool *in vivo* and the physiological relevance of this is not known. The measurement of serum concentrations of VEGF has therefore been speculated upon as being an artificial situation, which measures both the freely circulating VEGF and that released by platelets upon activation during the clotting process (Verheul *et al*, 1997). Therefore the significance of these studies that measure serum VEGF concentrations remains unclear, and plasma levels of VEGF may be a more accurate reflection of any biologically active circulating angiogenic cytokine. At present we have no information on the normal plasma concentrations of VEGF or if it varies during the menstrual cycle.

The aim of our study was to measure serum and plasma concentrations of VEGF in a group of healthy pre-menopausal volunteers and to determine if there is a cyclical variation during the menstrual cycle.

## MATERIALS AND METHODS

### Subjects

Twenty healthy pre-menopausal women with no significant past medical history were recruited to the trial. All had regular menstrual cycles and were not taking any form of extrinsic hormones, such as the oral contraceptive pill. Ethical Committee approval was sought and obtained for the study and all volunteers gave written informed consent.

### Collection of blood samples

Blood samples were taken from the patients for between 4–6 weeks depending on the length of the menstrual cycle. An average of eight samples was taken per patient (Range 6–9). These samples were drawn at 4 day intervals over the course of a single cycle. Samples were collected using the Vacutainer (Beckton Dickinson, UK) system, and approximately 20 mL of blood was drawn at each sampling interval. Samples were collected into three different tubes for analysis. Glass tubes containing SST Gel and Clot Activator were used to assay for FSH, LH, Oestradiol and Progesterone. Plasma samples were collected in glass tubes containing EDTA as an anticoagulant and were spun down at 10 000 r.p.m. for 10 min and then frozen at −70°C within 20 min of collection, pending analysis for VEGF. Serum samples were collected in plain glass tubes and allowed to coagulate for 60 min prior to spinning down and freezing at −70°C, again pending analysis for VEGF.

### VEGF determination

Plasma and serum samples were analysed for VEGF using a commercially available sandwich enzyme linked immunosorbent assay (ELISA) that was obtained from R&D Systems Europe, Abingdon UK (Quantikine Human VEGF). This kit has been used in many previous studies and has a low inter- and intra-assay error range (Intra-assay Error: 6.7–4.5 CV% in Serum/Plasma, Inter-assay Error: 8.8–6.2 CV% in Serum/Plasma). This assay is specific for VEGF and does not detect related molecules such as platelet derived growth factor or placental growth factor.

Briefly, a monoclonal antibody specific for VEGF 165 has been precoated onto the supplied microplate wells. Standards and samples are pipetted into the wells and the immobilised antibody binds any VEGF present. After washing away any unbound substances, an enzyme-linked polyclonal antibody specific for VEGF 165 is added to the wells. Following a further wash to remove unbound substance, a substrate solution is added to the wells and colour develops in proportion to the amount of VEGF bound in the initial step. The colour development is stopped after a specified time period with stop solution and the intensity of the colour is measured. A standard curve is constructed using supplied VEGF standards. All samples were assayed in duplicate and readings were made on a Dynatech MR5000 micro-plate reader with the test filter set at 450 nm and correction wavelength set at 570 nm, to account for optical imperfections in the plate. The sensitivity of the assay is 9.0 pg ml^−1^ as quoted by the manufacturer.

### Hormone determination

Samples were analysed using an automated Bio-Immuno-1 analyser, which gives direct readouts for FSH, LH, Oestradiol and Progesterone levels in serum.

### Determination of menstrual phases

Menstrual cycles for each patient were divided into follicular and luteal phases on the basis of the last recalled onset of menses, the actual date of menses during the study period, and an observed LH peak followed by a progesterone level that fell to within the luteal phase values of the assay used.

### Statistical analysis

The significance between VEGF concentrations, oestradiol and progesterone levels of the two phases of the menstrual cycle was calculated using the Mann–Whitney *U*-test for non-parametric data. Spearman's Correlation between the variables was also calculated. Statistical significance was set at a *P* value of less than 0.05. Calculations were performed using SPSS for Windows Version 7.5.1 (SPSS inc.).

## RESULTS

The median age of the enrolled volunteers was 36.3 years (range 28–47). The length of the menstrual cycles varied from 23–34 days with a mean cycle length of 27.8 days. All cycles were found to be ovulatory, as witnessed by an LH peak followed by an appropriate mid-luteal peak in progesterone levels.

Progesterone levels were significantly higher in the luteal phase of the cycle than in the follicular phase of the cycle (*P*<0.005), which would be consistent with the expected normal ovulatory luteal function. Oestradiol levels were significantly higher during the follicular phase of the menstrual cycle (*P*<0.005), and again these values correspond to the expected range of oestradiol in ovulatory cycles.

Serum VEGF levels were found to be significantly higher than plasma VEGF levels. The median value for serum VEGF throughout the cycle was 261.13 pg ml^−1^ (range 6.01–774.76, Standard Deviation 159.6) whereas the median value for plasma concentration of VEGF was 5.78 pg ml^−1^ (range 0.0 to 70.72, Standard Deviation 13.47), although these values for plasma are below the sensitivity of the ELISA kit as quoted by the manufacturer (sensitivity=9.0 pg ml^−1^). This difference was statistically significant with a *P*-value of less than 0.005 using the two-tailed Mann–Whitney *U*-test. The majority of the measurements for plasma levels of VEGF at all phases of the cycle were under the limit of detection of the VEGF ELISA kit.

**Figure 1:**
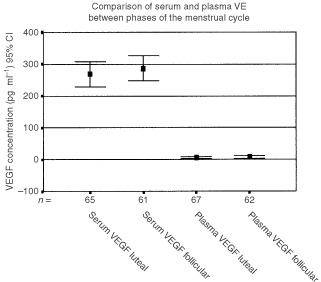
Comparison of serum and plasma VEGF concentrations in both phases of the menstrual cycle. Error Bars represent 95% C.I.

Samples were redefined into luteal and follicular phases based on an observed LH peak and re-analysed. We found no significant difference between either serum or plasma levels of VEGF in the luteal and follicular phases of the cycle (Wilcoxon Sign Rank test *P*=0.267 for serum levels and *P*=0.424 for plasma levels). With respect to serum levels of VEGF the mean level during the luteal phase was 268.8 (range 12.23–693.37, 95% Confidence interval 39.9). Mean level of serum VEGF during the follicular phase of the cycle was 286.66 (range 6.01–774.76, 95% Confidence Interval 38.97) (Figure 1

). We found no significant correlation between either plasma or serum levels of VEGF and either FSH, LH, Oestradiol or Progesterone levels using Spearman's correlation.

## DISCUSSION

Angiogenesis is essential for tumour growth and metastasis (Folkman, 1990). The angiogenic process is complex and is regulated by a wide variety of growth factors, both positive and negative. Vascular Endothelial Growth Factor (VEGF) is a potent and widely distributed angiogenic peptide, which has been widely implicated in tumour progression. This growth factor is a dimeric 34–42 Kd glycosylated basic protein, which is encoded in four molecular isoforms. The two larger isoforms (VEGF 189 and VEGF 209) remain cell associated whereas the smaller isoforms (VEGF 121 and VEGF 165) are secreted as soluble molecules. The latter isoforms induce their angiogenic effects by binding to the specific transmembrane tyrosine kinase receptors KDR/Flk-1 and Flt-1, which are selectively expressed on vascular endothelial cells. Higher expression of VEGF and its receptors in breast tumours has been shown to be significantly associated with high intra-tumoural micro-vessel density and also with poorer prognosis (Weidner, 1991; Bosari, 1992).

In addition to being upregulated within tumours, levels of angiogenic cytokines have been reported to be increased in the serum and other body fluids of patients with cancer (Yamamoto, 1996). In breast cancer patients, higher levels of serum VEGF have been demonstrated in patients with stage III as compared to stage I or stage II disease (Yamamoto, 1996).

While the significance of circulating levels of VEGF in cancer patients is now being elucidated, the significance of circulating VEGF levels in healthy volunteers is unclear. Serum levels of VEGF have been reported to be higher in post-menopausal volunteers than in pre-menopausal women (Heer *et al*, 1998) with a mean of 243.9 pg ml^−1^ (InterQuartile range 128.5–425.6) in post-menopausal women *vs* a mean of 165.0 pg ml^−1^ (IQR 88.2–236.3) in pre-menopausal women. This author also reported a significant difference in serum levels of VEGF between the two phases of the menstrual cycle in 14 healthy volunteers. A median value of VEGF during the luteal phase of 174.7 pg ml^−1^ was significantly lower than a level of 206.5 pg ml^−1^ (*P*=0.03).

Heer *et al* (1998) hypothesised that any possible survival advantage (reported by authors such as Badwe *et al* (1991) of patients who undergo breast cancer surgery in the luteal phase of the cycle could be due to this difference in serum levels of VEGF found in his study. However, Chung (1998), who also measured serum concentrations of VEGF in 14 healthy pre-menopausal volunteers, carried out a similar study. This study demonstrated no difference in serum VEGF concentrations between the two phases of the cycle.

Workers such as Banks demonstrated more recently that VEGF is contained in platelets and that this should be taken into account when measuring circulating VEGF concentrations. It is now apparent that many haematological cells are capable of releasing VEGF (Banks, 1998; Webb *et al*, 1998; Mohle *et al*, 1997). The extent to which these cells normally contribute to the circulating VEGF pool *in vivo* and the physiological relevance of this is not known. The measurement of serum concentrations of VEGF has therefore been speculated upon as being an artificial situation, which measures both the freely circulating VEGF and that released mainly by platelets upon activation during the clotting process. The actual act of collecting serum samples causes platelet activation, and release of large amounts of VEGF from the alpha granules contained within the platelets. It therefore remains unclear which measurement of circulating VEGF actually represents the true amount of biologically active VEGF in the circulation.

With respect to our study, we recruited 20 healthy pre-menopausal volunteers, and followed them for one menstrual cycle. This study is the largest to date, with both previous similar studies having only 14 patients enrolled. In our study both plasma and serum samples were collected, and this is therefore the first study to report on plasma values of VEGF during the menstrual cycle.

Menstrual phase was assessed by reviewing hormonal profiles. We found all cycles to be ovulatory in our group of volunteers. Normal hormonal patterns were also found. However we found no significant difference between phases of the menstrual cycle with respect to either serum or plasma concentrations of VEGF. The mean concentration of VEGF, which we found in serum, was 261.13 pg ml^−1^. This value is higher than the values quoted by Heer *et al* (1998) for both pre- and post-menopausal women (165 pg ml^−1^ and 243.9 pg ml^−1^ respectively). It is possible that this difference could be accounted for by a population difference, but we suspect that differences in sample collection is a more likely reason. In his study, Heer *et al* (1998) makes no mention of how samples were collected or his method for serum separation. Because we were measuring both serum and plasma samples, we were extremely vigilant in our sample collection. All samples were collected by identical means, and serum samples were allowed to clot for 60 min to ensure thorough platelet activation before centrifugation and freezing. This alone may account for our higher value for serum concentration of VEGF, although in addition, Heer *et al* (1998) only utilised three post-menopausal controls, and so we doubt that this sample can be relied upon to represent the population median. Other studies have quoted serum levels of VEGF in control populations to be similar to ours (Verheul *et al*, 1997; Webb *et al*, 1998), and these studies used similar sample collection protocols.

Plasma concentrations of VEGF in this study were much lower than serum concentrations (mean of 261 *vs* 5.8 pg ml^−1^), as would be expected if VEGF released from platelets contributes most of the VEGF concentration found in a sample of serum. Most of the plasma concentrations measured were below the quoted range of the ELISA kit (9 pg ml^−1^). These values are lower than previously quoted in previous studies quoting population concentrations of VEGF in plasma (46.4 pg ml^−1^ by Webb *et al*, 1998). Once more, the most likely reason for this was our meticulous collection method, which was designed to minimise platelet activation. A previous study by Banks (1998) recommended the use of glass EDTA tubes over plastic tubes to minimise platelet activation and this was borne in mind when our protocol was devised. In addition, the young age of our sample population could account for some difference. Vascular endothelial growth factor concentrations can be raised by all manner of pathological processes from trauma to liver disease to pre-eclampsia (Iitaka *et al*, 1998; Akiyoshi *et al*, 1998; Baker *et al*, 1995; Grad *et al*, 1998). In a population with an older mean age, you could reasonably expect to find a cross section of pathology that is not present in our study population, thus giving our lower plasma concentrations.

This study has demonstrated no difference in serum concentrations of VEGF during the different phases of the menstrual cycle in a group of healthy volunteers. We also demonstrated no obvious difference in plasma concentrations of VEGF between the phases of the cycle, but most of the measurements were below the level of accuracy reported by the ELISA kit manufacturer. With the sensitivity of this ELISA test, therefore, we must still regard the question of whether there is a variation in plasma concentrations of VEGF throughout the menstrual cycle as unanswered.

At present, therefore, there is no evidence to suggest that circulating levels of vascular endothelial growth factor vary with the phase of the menstrual cycle in healthy pre-menopausal women. We have confirmed that serum concentrations of VEGF are significantly higher than plasma concentrations, and that most of the plasma measurements are below the limit of the commercially available kit that we used.

Based on our results, we conclude that serum concentrations of VEGF do not vary with the menstrual cycle in healthy volunteers. Therefore if circulating VEGF were to be utilised in the future for a tumour marker, then timing of samples will not have to be altered to take menstrual phase into account. Variations in plasma VEGF may vary with the phase of the menstrual cycle, but most of the measurements in healthy volunteers are under the limit of detection of our ELISA, so we would have to regard this question as unanswered.
